# Supplementary material for: Cellular Effects of Selected Unsymmetrical Bisacridines on the Multicellular Tumor Spheroids of HCT116 Colon and A549 Lung Cancer Cells in Comparison to Monolayer Cultures
Source: Int J Mol Sci. 2023 Oct 30;24(21):15780. doi: 10.3390/ijms242115780 (PMC10649579; doi:10.3390/ijms242115780)
Supplement: Supplementary file 1 [file ijms-24-15780-s001.zip › ijms-2674150-supplementary.pdf]

International Journal of *Molecular Sciences*

MDPI

Supplementary materials

Cellular Effects of Selected Unsymmetrical Bisacridines on the Multicellular Tumor Spheroids of HCT116 Colon and A549 Lung Cancer Cells in Comparison to Monolayer Cultures

Jolanta Kulesza, Ewa Paluszkiewicz, Ewa Augustin*

Department of Pharmaceutical Technology and Biochemistry, Faculty of Chemistry, Gdańsk University of Technology, Gdańsk, Poland; jolanta.kulesza@pg.edu.pl; ewa.paluszkiewicz@pg.edu.pl; ewa.augustin@pg.edu.pl

* Correspondence: ewa.augustin@pg.edu.pl

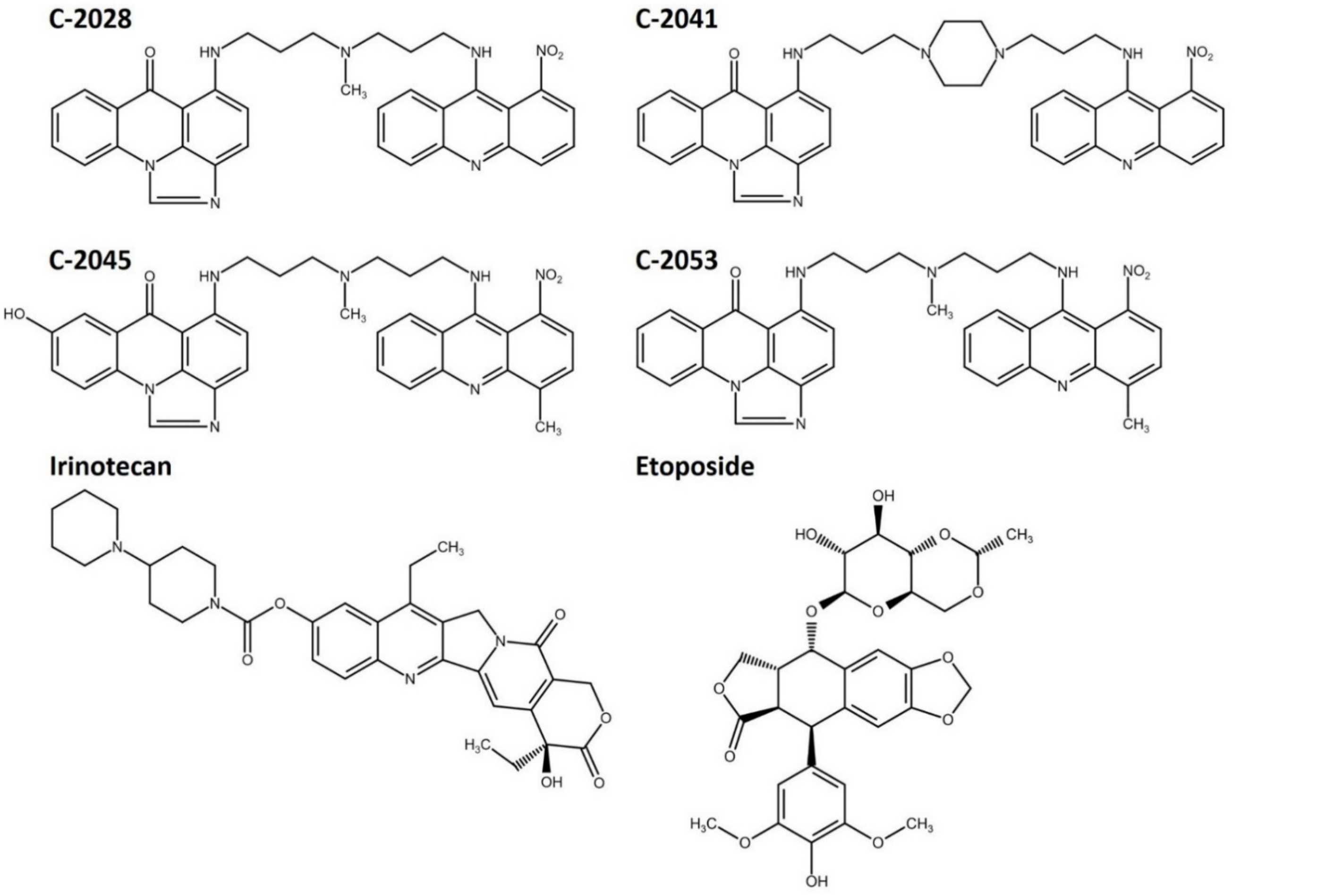

Supplementary Figure S1. Chemical structures of studied compounds: four unsymmetrical bisacridines (UAs) – C-2028, C-2041, C-2045, and C-2053 together with two reference compounds – irinotecan and etoposide.

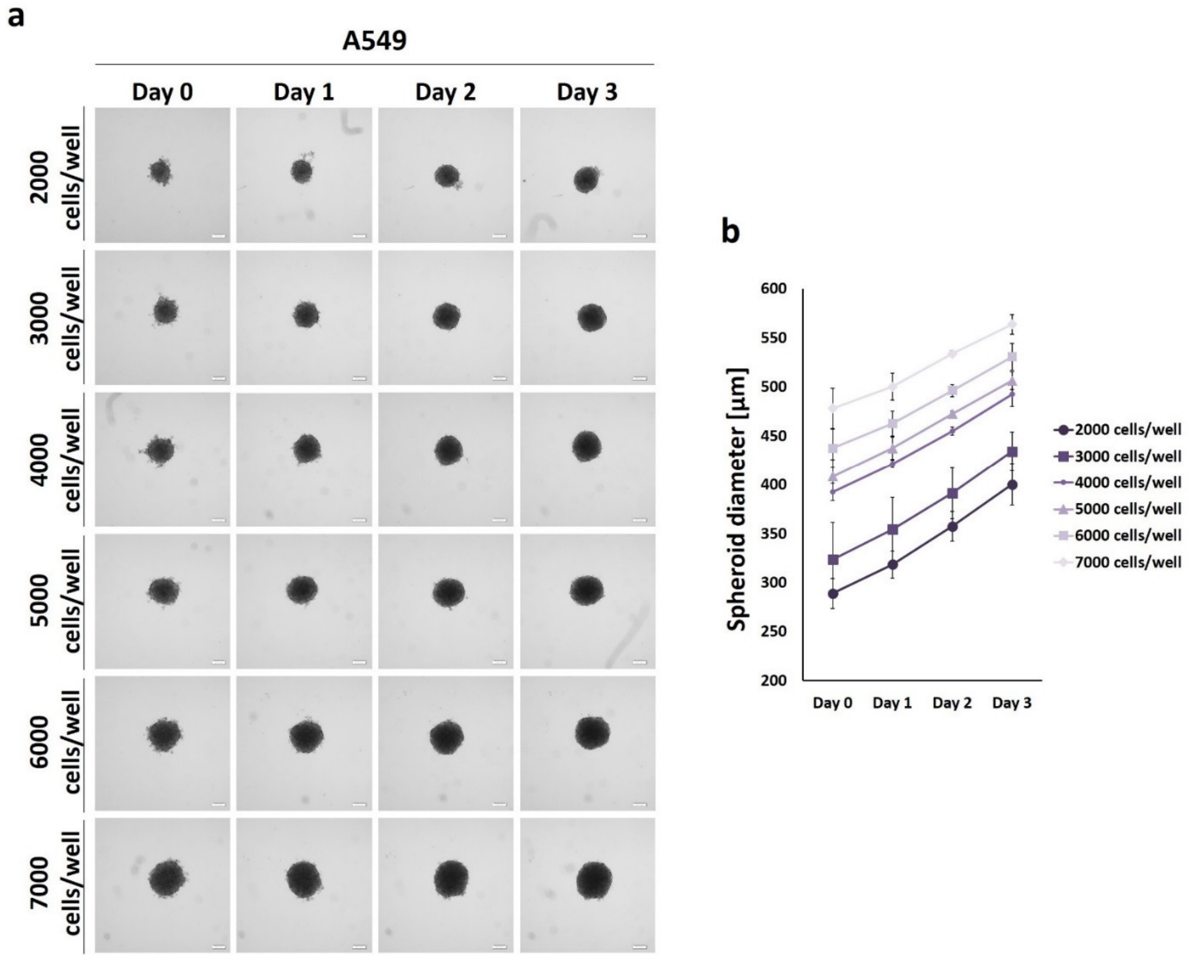

Supplementary Figure S2. Establishment of seeding conditions for A549 spheroid formation. Cell suspensions with different densities were seeded into ULA plates and incubated for 72h to allow spheroid formation. Then for 4 subsequent days images of spheroids were taken and diameters measured. **(a)** Representative microscopic images of A549 spheroids obtained from various seeding densities. **(b)** A549 initial tumor spheroid growth curves. Scale bar 200 µm. Values are mean ± SD. (n=3)
